# The Floral Tributes

**Published:** 1920-05-08

**Authors:** 


### The Floral Tributes.

Her Majesty Queen Alexandra sent a beautiful cross of narcissi, white orchids and flame-coloured carnations. On the card accompanying it were written the words in Her Majesty's own handwriting : " In ever grateful memory of Sir Henry Burdett, the most valued organiser of my Nurses' Pension Fund. May he rest in peace. Alexandra." The flowers from the Hon. Charlotte Knollys were sent " With deepest regret and regard for my dear old friend of many years." From The Nurses' Co-operation came a beautiful tribute in red and white geraniums. It represented the well-known badge of the Co-operation—the cross being in red and the circle in white; on the latter was a mauve ribbon with the words : "The Nurses' Co-operation" printed in silver. With this was a card bearing the following words : " In deepest regret for the loss of a great friend and benefactor." The Composing Staff of The Hospital and the *Nursing Mirror* at Messrs. Spottiswoode, Ballantyne & Co. sent a chaplet of red tulips, narcissi, white hyacinths, and palm leaves, and another chaplet of laurel leaves, lilies, mauve iris, and violets was from Mrs. Constance Wilcox (*Nursing Mirror*), " in honoured remembrance of her Editor-in-Chief." Personal arrangements had been made by the staff of the Scientific Press to send a wreath, but, in deference to the announcement, floral tributes were not generally sent. A beautiful cross of red roses and palm leaves from the children, grandchildren, and members of the family lay along the whole length of the coffin.

